# Sustainability and the real value of care in times of a global pandemic: SDG5 and Covid-19

**DOI:** 10.1007/s43621-021-00054-7

**Published:** 2021-10-18

**Authors:** Flora Ijjas

**Affiliations:** Department of Environmental Economics, Budapest University of Technology and Economics, Magyar Tudósok Körútja 2, Budapest, 1117 Hungary

**Keywords:** Sustainability, Reproduction, SDG, Care economy, Gender equality, Covid-19

## Abstract

The pandemic Covid-19 has been affecting the global population, causing profound social and economic problems. The aim of this paper is to analyse the health crises from an ecological economic and a gender equality perspective in order to see how the pandemic is affecting Sustainable Development Goal 5 (gender equality) and particularly Target 5.2 which is about eliminating domestic violence and 5.4 which is about the valuation of unpaid care and domestic work. Secondary data, facts and thoughts from scientific papers and other documents are being reviewed to understand our socio-economic systems’ effects on the reproduction of nature and on social reproduction. Results show, that capitalist systems exploit regenerative and reproductive workers of the demonetized economy, such as nature, unpaid caregivers, peasants, and indigenous gatherers. These exploitative systems also have contributed to the rise of the new pandemic Covid-19 by destroying natural habitats. The virus might have some short-term positive effects on the environment, but the backbone of society’s response is unpaid care work, therefore women are being exploited even more. Conclusions are, that patriarchal characteristics, such as normalized systematic domination and oppression of women (and other regenerative and reproductive workers) are on the long run a burden for the SDGs.

## Introduction

There is extensive research on environmental issues and gender inequality issues. The paper summarizes shortly the current state of knowledge of these issues but it’s main originality relies upon the review of papers covering the links between environmental problems and gender inequalities and upon the elaboration of those links by examining them throughout the case of the new pandemic Covid-19.

There are only a few articles and surveys about Covid-19’s effects on SDG 5 and no literature about the deep lying reasons behind those effects, to the best of my knowledge. Therefore, this paper seeks to answer following questions:

1. How is Covid-19 effecting SDG 5?

2. Is it endangering the implementation of SDG 5, particularly Target 5.2—eliminating violence against women and 5.4—the recognition of unpaid care work?

3. Is this process connected to sustainability and other SDGs, and if yes, than how?

In the first part main environmental concerns and existing gender inequalities from all over the world will be summarized shortly. Here, literature review has been used to introduce the global, major violations of nature and women today. After that, the case study of Covid-19 will be analyzed through ecological and gender equality lenses. The analysis is based on literature review about the effects of the pandemic on women and on SDG 5 and also on the pandemic’s connections with environmental degradation. The last part reviews and explains the theoretical background provided by several scientists about the systematic socio-economic exploitation of women and nature and elaborates the real value of care using logical argumentation.

Findings about the connections between the exploitation of the backbone of society’s response for the crises (which is paid and unpaid care work and other forms of metaindustrial labor) and the exploitation of natural resources might be of interest for economists, ecologists and sociologists as well and could reinforce deep structural change for a more safe and just world. That’s where the paper’s relevance lies.

## Environmental problems

The official United Nations’ (UN) definition of sustainability has three dimensions: environmental protection, economic development and social equity. For the environmental dimension Rockström et al. [1] showed with the ‘planetary boundaries’ concept that 9 processes regulate the stability and resilience of the Earth system. Society’s activities have already pushed 4 of these—namely climate change, biodiversity loss, shifts in nutrient cycles (nitrogen and phosphorus), and land use—beyond their boundaries, which increases the risk of generating possibly irreversible environmental changes. Beyond environmental problems, we have to face socio-economic problems as well. Responses like green technologies are problematic. The so called “rebound effect” shows that energy and resources saved by consuming environmentally friendly products can end up in producing other products for “greener” consumption. Also, wealthier societies’ growing consumption needs lead to greater poverty in low-income countries due to agroimperialist exploitation. Core countries have higher levels of domestic material consumption and higher physical imports, therefore sustainability has to be analysed globally in order not to miss important facts such as possible exploitation of periphery (and semi-periphery) countries for sustaining core countries’ consumption patterns [2]. Beside the regenerative and reproductive work done by nature, peasants, and indigenous gatherers in periphery countries, also the reproductive work done by unpaid caregivers (mainly women) is being exploited and needs to be integrated into the sustainability discussion.

The UN established in 2015 a roadmap for sustainable development, called the 2030 Agenda for Sustainable Development. It has 17 Sustainable Development Goals (SDGs) that attempt to cover all planetary boundaries and economic problems. But they also have a social dimension covering gender equality (SDG 5).

## The reality of women worldwide: gender inequalities and violence against women

An important part of the social equity dimension of sustainability is gender equality. It is a state, when access to rights or opportunities is unaffected by gender. High gender inequalities reduce women’s chances for a safe and just life. According to UN Women and the Department of Economic and Social Affairs [3] global gender equality is unfortunately still far on the horizon. Women and girls around the world are 4% more likely than men and boys to live in extreme poverty, and the risk rises to 25% for women aged 25–34. Women also had a 10% higher risk of experiencing food insecurity than men in 2018.

One in 5 women and girls between the ages of 15 and 49 have reported to have experienced physical or sexual violence by an intimate partner within a 12-month period. Forty nine countries currently have no laws protecting women from domestic violence. Seven hundred and fifty million women and girls were married before the age of 18 and at least 200 million women and girls in 30 countries have undergone some form of female genital mutilation. Only 57% of women married or in a union freely make their own decisions about sexual relations, contraceptive use and reproductive health care. Three in 4 human trafficking victims are women and girls [3].

An estimated 15 million girls compared to 10 million boys of primary-school age are out of school. Women spend three times as many hours each day in unpaid care and domestic work than men. Based on data from 2018, only 19% of all countries had a comprehensive system to track budget allocations for gender equality. In 18 countries, husbands can legally prevent their wives from working; in 39 countries, daughters and sons do not have equal inheritance rights. Women are just 13.8% of agricultural land holders. Women and girls are responsible for water collection in 80% of households without access to water on premises, according to data from 61 developing countries. Women’s representation in national parliaments at 23.7 per cent is still far from parity [3].

According to the World Employment Social Outlook 2016 [4] women are concentrated at the bottom of the global value chain, they have the lowest paid jobs and insecure forms of self-employment whilst often having no access to decent work and social protection.

According to the European Union’s (EU) gender equality database^1^ the pay and pension gap in the EU remains and doesn’t show any signs of narrowing. In 2014 women were still paid 16.7% less and women’s pension was 40% lower on average. In 2019 women accounted for 31% of parliament members. The number of women in business leadership was low, women accounted for just 27.8% of board members of the largest publicly-listed companies. The new gender equality strategy of the European Committee^2^ has been released on March 5th this year. The strategy is lacking insight regarding the main cause of inequality, namely men not doing their part of care work. The strategy’s attempts to solve the issue, seem to only sustain the global care chain.

The data above shows, that gender inequalities persist in most of the countries. The new pandemic Covid-19 hits countries worldwide and it puts a great pressure on the care economy, on our health care systems and on our unpaid care workers who are mostly women.

## Methodology

The research questions (How is Covid-19 effecting SDG 5? Is it endangering the implementation of SDG 5? Is this process connected to other SDGs, and how?) cover an interdisciplinary field. It is difficult to use rigorous scientific methods to prove hypotheses on a partly moral (equality), partly socio-economic, partly ecological topic, when we don’t want to lose important information. Therefore, qualitative research methods: literature review, a case study with ecological and gender equality analyses and secondary data analyses with logical argumentation have been chosen as methods that could best serve the answering of the research questions and prove hypotheses:

- Hypothesis I. The pandemic is endangering the implementation of SDG 5, particularly Target 5.2—eliminating violence against women and Target 5.4—the recognition of unpaid care work.
- Hypothesis II. The exploitation of the backbone of society’s response for the crises (which is paid and unpaid care work and other forms of metaindustrial labor) and the exploitation of natural resources are interconnected.

It is a descriptive, explanatory research that aims to explore how Covid-19 is affecting SDG5, which is an under-researched area. The paper also explores whether or not there is a connection between the exploitation of women and nature and it wanted to search for explanations if the answer is yes.

The case study method has been chosen because the case of the Covid-19 pandemic highlights the exploitation of reproductive work, as the hierarchical structure of productive vs. reproductive work might become more obvious, when everyday life’s practical problems manifest themselves as a result of the pandemic, and as theory can be more easily translated into practice.

The paper is also conducting a deductive research aiming to test the theory of capitalist patriarchy and the care economy (used in feminist studies, feminist economics and ecofeminism) by analyzing the case of the global pandemic from ecological and gender equality lenses.

The last part reviews and analyses the theoretical background about the systematic socio-economic exploitation of women and nature and elaborates the real value of care using logical argumentation.

## Limitations

The links between ecological economics and feminist economics and also their criticism could be further elaborated but hasn’t been covered in the scope of the paper. As we don’t know yet, what exact consequences Covid-19 is going to have in the longer run, it is a limitation for the results as well. Also, it is for further research to find out, why patriarchal values and gender stereotypes are so firmly entrenched.

## The case of Covid-19

The coronavirus disease 2019 has been declared a pandemic by The World Health Organization (WHO).^3^ As of 26 March 2021, 1,25,614,782 global cases have been identified with a total of 27,57,339 deaths.^4^ This data comes from 192 countries, which means that the whole population is affected in some way by Covid-19. Ecological economic and feminist economic literature review might help to show possible reasons for and effects of this new pandemic.

### Ecological perspective: the role of biodiversity loss and intensive agriculture in animal-borne diseases and Covid-19

Some of the processes that violate nature could have possibly supported the Covid-19 pandemic. Jones et al. [5] identified 335 diseases that are linked to environmental change causing human behaviour. At least 60% of these came from animals. Deforestation, destruction of natural habitat, causing erosion by building roads and houses, by mining, etc. bring people into closer contact with animals and also degraded habitats are likely to carry more viruses and most of the farming practices also contribute to the emergence of these diseases [5]. Rohr et al. [6] has found, that intensive agriculture contributed to more than half of all infectious diseases, that leaped from animals to humans since 1940.

Loss of biodiversity and deforestation lead to risking a very important ecosystem service, that is the ability to reduce virus emergence. Diversity within species and natural habitats supports living beings’ adaptive capacity against viruses and bacteria. If in the agricultural production process corporations and nations only breed a few species in extreme high numbers, while caused the fast extinction of other species (and the destruction of habitats and erosion)—the ability to reduce virus emergence will be put at risk. Animal agriculture is one of the largest contributors to greenhouse gases emissions and it provides an amazing opportunity for viruses to thrive.^5^

We urgently have to deal with the contradictions of intensive agriculture, that on the one hand provides more food for the population but on the other hand is unacceptable from an animal ethics perspective and that also threatens more planetary boundaries (such as the biosphere integrity, the biogeochemical cycles, the land-systems, and the fight against climate change).

However, the Covid-19 pandemic has some positive impacts on these planetary boundaries as well. It might cause a drop in emissions in the short run. In the long run emissions will probably rebound, but might remain down until a vaccine is found. On the other hand, the emerging economic crises might also keep emissions depressed for some years. Some of the new adaptive behaviours, business models and technologies could also support the transition to a low-carbon economy. In most core and some semi-periphery countries home-schooling, home-working and remote medical services might lead to an increase in flexible working hours. If public transport demand was flat across the day, then public-transport investments could be more cost-effective.^6^

Sustainability is not just about environmental protection. Sustainability has three dimensions. One of them is social equity. Domestic violence, various forms of male chauvinism, sexual violations of children throughout the internet could and already are escalating amid curfew and lockdowns both in developed and in poorer economies [7].

### Gender equality perspective on Covid-19

#### Effects on SDG 5, Target 5.4—unpaid care work

The 2020 new corona virus pandemic shows, how fragile our economic and health care systems are. In 18 countries women are doing much more around the family and the house due to Covid-19 prevention measures [8]. In Hungary men had been doing more household work, but women carried out a far greater proportion of child-related tasks prior to the introduction of the coronavirus-related regulations [9].

The unpaid care work, that is mostly done by women is the backbone of the response to Covid-19. This work consists of maintaining the health and well-being of children, the sick and the elderly as well as health promotion and prevention activities. As a result of school and day-care closures some mothers will lose, or “willingly” give up their jobs to stay home with the children. If they can work in home-office, then they face stress and multitasking, that might affect their health. Grandmothers also provide great help, possibly risking their own health. Low-paid care work jobs are also mostly done by women, who now will be more exposed to the risk of contracting the disease. The rise in unpaid care work has also other consequences for gender equality: the psychosocial effects from providing care to infected relatives. Community health workers (mostly women) of periphery countries will be needed as well, but they will keep receiving little or no compensation for their work [10]. Easter European and Central Asian organizations urged the international community and political decision-makers to recognize the risks of Covid-19 for women: it could result in a roll back in women’s rights, if appropriate responses and recovery planning are not urgently undertaken [11].

#### Effects on SDG 5, Target 5.2—violence against women

According to von Werlhof [12] patriarchy has the characters of systematic domination and oppression of women. Now, in the times of Covid-19 many women have to bear with their often abusive male partners within the four walls of their homes, a black box in neoclassical economics. On the 6th of April 2020 UN Secretary-General António Guterres has warned about a sharp rise in domestic violence (in some countries—also in Hungary—the number of women calling support services has doubled^7^) amid global coronavirus lockdowns, that threatens SDG 5, Target 5.2 that is the elimination of all forms of violence against all women and girls. The rise of violence was expected as men restricted to their homes are having more time to do what society allows them to do: show and practice domination. Curfew also makes it easier for abusers to isolate their victims. China and the UK reported, that domestic violence have more than doubled in Covid-19 lockdown period between February and April 2020, compared to the same period of the previous year [13]. The Italian national network of shelters for women marked a steep increase (74.5%) in the number of women who contacted shelters within the period of Covid-19 lockdown [14]. Belizzi et al. [14] even states that “Covid‐19 and violence against women are interrelated pandemics”. According to Bellizzi et al. [15] Covid-19 disruptions have unfortunately also lead to “significant delays in programmes to end FGM”, which is female genital mutilation, “potentially leading to around two million more cases of FGM over the next decade” and to “an expected additional 31 million cases of gender-based violence and 13 million more child marriages over the next 10 years”.

A new UN Women survey [16] assessed the pandemic’s effects on SDG 5 in 11 Asia–Pacific countries. Data showed that “women’s economic resources are being hit hardest”. It also states, that the pandemic has disproportionately affected women’s mental and emotional health and as the unpaid care and domestic workload has increased, “women are bearing the heaviest burden”. Data showed, that the salary of women has fallen caused by reduced formal workers’ hours and lost informal workers’ jobs. The survey also showed that the curfews had put women’s safety at risk, and that “institutional responses are inadequate”. It’s main finding is, that Covid-19 is putting the implementation of SDG 5 at risk in Asia and the Pacific region.

After reviewing the data, that exist upon the gendered effects of Covid-19, we can see that there are many negative effects for women all over the world (UK, China, Hungary, Asia–Pacific countries, etc.). In the next section following questions will be discussed: why is Covid-19 affecting women negatively and how is this process also linked to environmental degradation. For that purpose some fundamental concepts such as social and natural reproduction and capitalist patriarchy and production will be described, and used as axioms.

## The reasons behind Covid 19’s negative effects on SDG 5—hierarchical division between “production” and “reproduction”

### Theoretical background about the care economy

According to the International Labour Organisation [17] women carry out at least two and a half times more unpaid household and care work than men do.

The Australian Bureau of Statistics recorded [18] that the figure for the individual, replacement cost of unpaid labour was 43.5% of GDP. The housekeeper replacement cost was 41.6% and the opportunity cost method recorded an equivalent of 57.1%t of GDP. Economic Nobel prize nominee Waring cited a 2017 Price Waterhouse Cooper research^8^ in one of her presentations showing that the largest economic sector in Australia is unpaid childcare, and the second-largest sector is all the rest of the unpaid work. These sectors are bigger than banking, insurance and financial intermediation services combined. According to Waring [19] activities with strong gender dimensions were, and still are excluded from UNSNA rules. These are: cleaning, repair of household durables, preparation and serving of meals, care, training and instructions of children, care of sick and inform people, and the transportation of member of the households and their goods.

These forms of care work can be aggregated into one single term, that is of a great importance in our discussion. It is the so called ‘social reproduction’. Katz [20] says that social reproduction is “the broad material social practices and forces associated with sustaining production and social life in all its variations” (pp. 18). Ruder and Sanniti [21] adds that it can mean child care, elderly care, teaching, household work, cooking, cleaning and also water collection. This work is largely unpaid although it is one of the biggest economic sectors, it is naturalized, feminized and racialized [21].

As Fraser [22] points out, if we want to challenge gender domination, than “the institutionalized separation of two supposedly distinct kinds of activity: on the one hand, so-called “productive” labour, historically associated with men and remunerated by wages; on the other hand, “caring” activities, often historically unpaid and still performed mainly by women” need to be taken into account. Fraser assumes, that “this gendered, hierarchical division between “production” and “reproduction” is a defining structure of capitalist society and a deep source of the gender asymmetries hard-wired in it”.

### Gender and nature domination of capitalist patriarchy

Ruder and Sanniti [21], McMahon [23] Katz and Kirby [24] and Green [25] use the term capitalist-patriarchy as a socio-economic system, that is based on the limitless ‘resourcing’ of women and nature. These researcher state, that this concept is relevant to sustainability research and ecological economics, because it is exploiting nature’s resources as well. Ruder and Sanniti [21] describe the term as the manifestation of dominance over women and nature. They highlight, that gender biased and anti-ecological structures of power in the capitalist-patriarchy are grounded in the denial of dependency and interdependency. Werlhof [12] claims, that patriarchy and capitalism have similar characters such as war (to plunder and conquer); systematic domination; oppression of women; class divisions; systems of exploitation of humanity and nature; ideologies of male ‘‘productivity’’ and religions of male ‘‘creation’’ and states that these patriarchal characteristics have been dominating Europe in the last 5000–7000 years. Werlhof also suggests, that capitalism aims to normalize domination and to realize a world without nature or mothers therefore without dependence. According to McMahon [13] capitalist patriarchy disguises “the ways in which the market and economic man are dependent on unsustainable transfers from nature and from unpaid work” (pp. 168).

A socio-economic system that has patriarchal-capitalist characteristics contributes to the domination over women and nature. Salleh [26] points out: “North and South, East and West, the flexible, do-it-yourself, cooperative economy of women is daily subsumed by private and public spheres alike; just as the degraded ‘resource base’ of nature silently absorbs the longer-term costs of what is called ‘development’” (pp. 319).

Fraser [27] suggests that the expropriation of social and natural reproduction (and as a result gender and racial oppression, ecological crisis, underemployment and unemployment, etc.) is a structural feature of capitalism. According to Moore [28] the historical and structural condition, of capitalist valorisation, is the undervalued “socially necessary unpaid labour” which is mostly women’s domestic labour, and the unpaid labour of nature such as fossil fuels. Malm [29, 30] even suggests that the so-called fossil economy, was not motivated by the cheapness of coal but by the possibility of labour domination [31].

It seems, that capitalist production relies on social reproduction and regeneration. This can also be called according to Salleh [32] “the reproduction of the humanity-nature metabolism”, or on “metaindustrial labour”. These types of labour done by women, peasants, indigenous gatherers are absolute necessities for the global economy to thrive. These types of work mostly have a cyclical logic that helps to sustain cycles of nature as well. Salleh [33] points out, complexity theory and multidisciplinary methods have been being around already for a long time, mainly applied by mothers. Mothers have always been precautious instead of taking risks. They are trying to be cooperative in order to balance financial sources with needs and they define development in terms of health and sustainability. According to the literature review, this type of work should be definitely valued more. It could be applied as ‘know-how’ when building a new “just and safe” world.

## Findings

I have found that the case of Covid-19 highlights the problem of exploitation of reproductive work, such as the exploitation of the reproductive work of nature and the exploitation of care work done by women. Results show, that the rise of the new pandemic Covid-19 could be an effect of destroying natural habitats, and even if it had short-term positive effects on the climate and on biodiversity; it has a significant negative one on gender equality.

Main results are:

1. The case of Covid-19 highlights the undervaluation of care work, which endangers UN Sustainable Development Goals, and SDG 5, Target 5.4 but also Target 5.2 in particular and therefore negatively affects the well-being of women (see Sect. “6.2.2”).

2. There is a connection between the exploitation of the backbone of society’s response for the crises (which is paid and unpaid care work and other forms of metaindustrial labor) and between the exploitation of natural resources. There is a deep routed belief system, that supports patriarchal values and therefore sustains domination of women (see Sect. “7.2”). This system is deeply rooted in capitalist structures, such as law-making, educational and environmental institutions and policymaking.

3. Furthermore, the case of Covid-19 highlights the role of manmade biodiversity loss and intensive agriculture in the appearance of animal-borne diseases, where for both of these processes, capitalist patriarchal socio-economic systems can be made responsible. I’ve also found, that new forms of Covid-19 adaptive behaviors are exacerbating the risk of violence against women, but on the other hand, these new behaviors and the economic slowdown have also resulted in a fall in emissions on the short run (see Sect. “6.1”).

## Discussion

Degraded habitats (caused by deforestation, destruction of natural habitat, causing erosion by building roads and houses, by mining, etc.) are likely to carry more viruses and most of the farming practices also contribute to the emergence of diseases. This could mean, that our socio-economic system has contributed to the new pandemic Covid-19. However, the results also show, that prior to Covid-19 prevention measures new socio-economic behaviours are on the rise, such as home-schooling, home-office and remote medical services and that some of these will have a rather positive effect for the natural environment, such as: drop in emissions due to less transport caused by less tourism, less business travel, shorter supply chains; drop in emissions due to the flattening of daily transport demand; drop in emissions due to less electricity consumption; public demand in more green surroundings and more clean air.

However, these new behaviours seem to have a rather negative effect on reaching SDG 5, such as: rise in domestic violence; more care work due to children and partners being at home; more care work due to sick and elderly people having only remote medical services and therefore a drop in the physical and psychological health of women.

A discussion about the possible effects of the new behaviours seems to be necessary, as on one hand we may have less emissions consequently, that would be of a great help in dealing with the delicate situation of the “climate change” or the “biodiversity loss” planetary boundary. But, there is the perspective of gender equality. The consequences of Covid-19 such as loss of income but also the new adaptive behaviours (such as home office) seem to lead to a worsening in women’s and children’s well-being caused by, for example the rise in domestic violence and therefore might put back the implementation of SDG 5, Target 5.2, that is the elimination of all forms of violence against all women and girls.

Results from the review of several papers show that the very idea that “the economic men” can be independent, he can be free of caring, he can do it all on his own—leads to serious consequences and it starts to show in the consequences of the pandemic, in the consequences of crossing planetary boundaries, such as in the consequences of climate change. The real value of health care system workers (such as doctors, scientists and public health officials) but also the value of low-wage service workers (such as people and mainly women working for retails and deliveries, cleaning services, home health assistance, garbage disposal, transport, etc.) showed itself in times of Covid-19 to economic decision-makers. As the backbone of the social response is unpaid care work—women are being exploited even more which is sustained by patriarchal characteristics, such as normalized systematic domination and ideologies of male “productivity”.

## Conclusions

Social reproduction done by women seem to be exploited in a similar way, as natural resources are being exploited. The link between the two processes is being made by our social and economic systems, that is capitalistic and patriarchal. This connection might be interesting for economists, ecologists and sociologists and could be used for further research.

The analogy between the exploitation of nature and women might be easily detected but to accept it, to take it seriously and to stop it, seems to be more difficult as long as patriarchal values dominate economic and social systems. A change in personal and cultural belief systems, a change in law-making and structural institutional change, eventually deep structural transformation is needed. Therefore, the findings might appeal to policymakers as well. Findings and their application in policymaking would bring significant change for the recognition of unpaid care work and for the elimination of violence against women. Therefore, conclusions of the paper could positively affect the life of many.

The 2030 Agenda for Sustainable Development has amongst its 17 Sustainable Development Goals (SDGs) the Goal of Gender Equality. It is supposed to be integral to all of the goals as the following motto suggests: “only by ensuring the rights of women and girls across all the goals will we get to justice and inclusion, economies that work for all, and sustaining our shared environment now and for future generations”. The Agenda 2030 also has the motto of “leaving no one behind”. Women working in the care economy mostly for free, providing the bulk of care in times of the Covid-19 pandemic are certainly among the ones who are continuously being left behind and therefore reaching SDG 5, might get even farther away, unless science and policymaking starts to work on a deep structural transformation, that makes gender equality and sustainability more realistic.

## Footnotes

1 https://eur-lex.europa.eu/legal-content/HU/ALL/?uri=CELEX%3A52016DC0739.

2 https://ec.europa.eu/info/policies/justice-and-fundamental-rights/gender-equality/gender-equality-strategy_en.

3 https://www.who.int/dg/speeches/detail/who-director-general-s-opening-remarks-at-the-media-briefing-on-covid-19---11-march-2020.

4 https://www.arcgis.com/apps/opsdashboard/index.html#/bda7594740fd40299423467b48e9ecf6.

5 https://hvg.hu/elet/20200422_eghajlatvaltozas_karosanyag_kibocsatas_fold_napja.

6 https://about.bnef.com/blog/covid-19-the-low-carbon-crisis/?fbclid=IwAR0QAkr-TjQ2nvdfStOwswOhd_mZ92WRX9cfNhX-3yz97WSDs-EY-uqE00U.

7 https://index.hu/belfold/2020/05/19/parkapcsolati_eroszak_csaladon_beluli_koronavirus_kijarasi_korlatozas_isztambuli_egyezmeny/.

8 https://www.pwc.com.au/australia-in-transition/publications/understanding-the-unpaid-economy-mar17.pdf.
